# Virtual Non-Iodine Coronary Calcium Scoring on Photon-Counting CT: Patient- and Plaque-Level Analysis ^†^

**DOI:** 10.3390/diagnostics16040599

**Published:** 2026-02-17

**Authors:** Müjgan Orman, Deniz Alis, Mehmet Onur Önal, Mustafa Ege Seker, Ahmet Akyol, Cem Alhan, Ercan Karaarslan

**Affiliations:** 1 Acibadem Healthcare Group, Department of Radiology, 34140 Istanbul, Türkiye; mujganyildizorman@gmail.com; 2 Department of Radiology, School of Medicine, Acibadem Mehmet Ali Aydinlar University, 34638 Istanbul, Türkiye; ercan.karaarslan@acibadem.edu.tr; 3 Department of Radiology, Cerrahpasa Faculty of Medicine, Istanbul University–Cerrahpasa, 34320 Istanbul, Türkiye; mehmet.onal@iuc.edu.tr; 4 Department of Radiology, University of Wisconsin–Madison, Madison, WI 53706, USA; smustafaege@gmail.com; 5 Department of Cardiology, School of Medicine, Acibadem Mehmet Ali Aydinlar University, 34638 Istanbul, Türkiye; ahmet.akyol@acibadem.com; 6 Department of Cardiovascular Surgery, School of Medicine, Acibadem Mehmet Ali Aydınlar University, 34638 Istanbul, Türkiye; cem.alhan@acibadem.com

**Keywords:** photon-counting CT, coronary calcium, virtual non-iodine, Agatston, risk reclassification

## Abstract

**Background/Objectives**: Whether PCCT-derived virtual non-iodine (VNI) images can replace true non-contrast (TNC) for coronary artery calcium scoring (CACS) remains uncertain, particularly for small, low-density plaques. We aimed to evaluate agreement between VNI and TNC for CACS at the patient and lesion levels and to quantify risk-category reclassification. **Methods**: In this retrospective single-center sample (May 2024–May 2025), 211 patients without prior coronary intervention and with nonzero CAC on TNC underwent PCCT. VNI (55 keV, QIR 1; 60 keV, QIR 4; PureCalcium) and TNC were reconstructed with matched section thickness/increment and kernel. Agatston and total calcified volume were recorded. Paired comparisons used Wilcoxon tests; reclassification across CAC categories (0, 1–99, 100–399, ≥400) and lesion-level false negatives (FNs) were assessed with TNC as the reference. **Results**: Low-keV VNIs (55–60 keV) underestimated CAC versus TNC. The median Agatston score decreased from 35.9 (IQR, 10.3–121.2) on TNC to 23.6 at 55 keV (*p* = 0.0006) and 22.2 at 60 keV (*p* = 0.0003); the total volume declined from 37.8 mm^3^ to 20.2 mm^3^ (*p* = 0.001) and 18.3 mm^3^ (*p* < 0.0001), respectively. More than half of patients were reassigned to a lower CAC category; despite no patient being CAC = 0 on TNC, 46.9% (55 keV) and 47.4% (60 keV) were labeled CAC = 0 on VNI. Because this study deliberately included only patients with nonzero CAC on the TNC reference, these CAC = 0 rates on VNI represent misclassification within a CAC-positive sample and should not be interpreted as population-level prevalence. At the lesion level, 95% of patients had ≥1 FN plaques (430 FN plaques total), typically small (median 8 mm^3^) and of low density (median Agatston 6). **Conclusions**: In this single-center sample with relatively low-burden calcification, low-keV VNI (55–60 keV) significantly underestimates CAC and down-classifies patients, with frequent “false-zero” assignments (defined as CAC_VNI = 0 despite CAC_TNC > 0) driven predominantly by small, low-density plaques.

## 1. Introduction

Over the past decade, coronary CT angiography (CCTA) and coronary artery calcium scoring (CACS) have become foundational tools for detecting and managing coronary artery disease (CAD), with robust evidence and professional guidelines supporting their prognostic value and clinical utility [1,2]. In routine practice, CCTA protocols typically include an electrocardiogram-gated true non-contrast (TNC) CT acquisition for CACS, followed by contrast-enhanced coronary angiography [3,4]. This standard approach separates the quantitative assessment of calcified plaque burden from the contrast-enhanced evaluation of the coronary lumen and plaque morphology, but it also requires an additional acquisition beyond the angiographic scan [3,4].

Photon-counting CT (PCCT) is an emerging detector technology that registers individual X-ray photons and bins them by energy. Compared with energy-integrating detector systems, PCCT offers improved spatial resolution and spectral separation and reduces electronic noise, enabling routine spectral reconstructions and multi-material decomposition [5]. These features have direct relevance for cardiac CT applications, where spectral information can be used to generate multiple image types from a single contrast-enhanced acquisition and potentially streamline established workflows [5].

Leveraging intrinsic spectral data, PCCT enables virtual non-contrast (VNC) images and virtual non-iodine (VNI) reconstructions from contrast-enhanced CCTA without an additional non-contrast scan. VNC aims to approximate TNC appearance, whereas VNI specifically removes iodine while preserving calcium signal, thereby supporting CACS estimation from a single contrast-enhanced CCTA dataset [3,6]. Comparative PCCT studies report that VNI generally shows closer agreement and fewer risk-category misclassifications than VNC, although neither method fully replaces TNC across all patients and scenarios. Reported performance depends on technical and lesion-level factors (e.g., tube potential, reconstruction settings, and calcium density), and very small calcifications remain challenging [3,4,7].

Importantly, VNI-based CACS behaves differently from conventional TNC-based CACS and may require parameter optimization. Prior studies have commonly reported the best correlation and agreement with TNC at 55 and 60 keV, supporting these monoenergetic levels as practical candidates for VNI-based calcium scoring [8]. However, most published evidence has been derived from European and North American samples, and external validation across different healthcare settings and populations remains limited [3,4,8]. In addition, most evaluations emphasize scan- or patient-level agreement metrics, whereas per-plaque detection analyses and artery-level localization of false-negative calcifications have been less frequently reported [3,4,9].

In this context, our study evaluated the accuracy of PCCT-derived VNI reconstructions at 55 and 60 keV for CACS, using TNC as the reference. Conducted in a tertiary private center within a middle-income Mediterranean country, our analysis prespecified (i) patient- and plaque-level agreement, (ii) systematic characterization of false-negative plaques (diameter, density, location), and (iii) risk-category reclassification.

## 2. Materials and Methods

### 2.1. Patients/Study Design

This retrospective, single-center study included all consecutive adult patients who underwent clinically indicated CCTA on a PCCT scanner between May 2024 and May 2025. Per institutional clinical practice, patients with contraindications to iodinated contrast administration did not undergo CCTA and, therefore, were not eligible for inclusion.

The Institutional Review Board approved this retrospective study and waived the requirement for informed consent for retrospective analysis of medical data. Patients with prior coronary interventions (coronary stent implantation or coronary artery bypass grafting; *n* = 25) and those without detectable coronary artery calcium on the TNC scan (*n* = 343) were excluded. Except for these exclusions, all consecutive adult CCTA examinations were included regardless of heart rate, heart rate variability, or body mass index.

### 2.2. CT Acquisition Protocol

All CT acquisitions were performed on a first-generation, dual-source PCCT system with quantum technology (NAEOTOM Alpha, Siemens Healthineers, Forchheim, Germany) equipped with two photon-counting detectors and 144 × 0.4 mm collimation. Scans were performed according to Society of Cardiovascular Computed Tomography guideline-based clinical practice.

TNC acquisition for coronary artery calcium scoring was performed in the same session immediately before contrast-enhanced CCTA, using ECG triggering in diastole. The TNC scan used 120 kVp with automatic tube-current modulation (IQ level 19), a 0.25 s rotation time, and a 3.0 mm slice thickness with a 1.5 mm increment.

The CCTA acquisition used 120–140 kVp (IQ level 80) and a 0.25 s rotation time. No routine beta-blocker premedication was administered per institutional protocol described previously [10]. Sublingual nitroglycerin spray was administered routinely unless contraindicated.

Acquisition mode selection was based on heart rate and rhythm. TurboFlash high-pitch mode was used for patients with a resting heart rate ≤ 70 bpm. Prospectively, ECG-gated sequential acquisition was used otherwise, with the acquisition window spanning 35% to 75% of the R–R interval. In patients with arrhythmia (including atrial fibrillation or frequent ectopy), a retrospectively ECG-gated spiral acquisition was performed.

All scans were obtained during a single end-inspiratory breath-hold, with z-axis coverage from the level of the carina to the diaphragm. Iodinated contrast medium (iohexol, 350 mg I/mL; Omnipaque, GE Healthcare, Princeton, NJ, USA) was administered as 70 mL followed by a 30 mL saline chaser (0.9% sodium chloride; Turk İlaç ve Serum Sanayi A.Ş., Ankara, Türkiye), injected at 6 mL/s through an 18G antecubital venous catheter using a dual-syringe power injector (Medrad Stellant; Bayer, Leverkusen, Germany). Contrast enhancement was controlled using bolus tracking with a region of interest placed in the descending aorta and a trigger threshold of 100 Hounsfield units.

### 2.3. CT Image Reconstruction

To keep the reconstruction conditions constant across series, all reconstructions used a soft-tissue kernel (Qr36) with a 3.0 mm slice thickness and a 1.5 mm increment. Reconstructions and postprocessing were performed using syngo.via (Siemens Healthineers), version v60. For calcium scoring, the reference standard was a TNC acquisition, reconstructed as a 70 keV VMI and a quantum iterative reconstruction (QIR) set to 0. We selected this reconstruction approach to align with published PCCT calcium-scoring methodology [3,4,7,8,9]; because PCCT is intrinsically spectral, reconstructing TNC in VMI space provides a standardized reference for comparisons across spectral reconstructions.

For each patient, two additional VNIs (PureCalcium) datasets were generated from the contrast-enhanced acquisition at 55 and 60 keV using the vendor’s spectral software, yielding three datasets per patient: (i) TNC reference (70-keV VMI), (ii) VNI-55 keV VMI, and (iii) VNI-60 keV VMI. The VNI reconstruction settings were selected based on prior PCCT optimization work identifying 55 keV (QIR 1) and 60 keV (QIR 4) as the best-performing configurations for agreement with TNC calcium scoring [11].

### 2.4. Coronary Artery Calcium Scoring

CACS was quantified with the Agatston method using a threshold of 130 Hounsfield units and a minimum connected area ≥ 1 mm^2^ [3,7,12]. For each patient, we recorded the Agatston score, total calcified plaque volume (mm^3^), and plaque count on all three series. For each calcified lesion present on the reference scan, detection on both VNI was adjudicated to identify false-negative lesions [7].

A board-certified radiologist with >5 years of dedicated cardiovascular CT experience (D.A.) reviewing approximately 10 cases per day in practice performed side-by-side comparisons across series, confirmed or corrected software-identified calcifications as needed, assigned lesions to major coronary arteries (LM, LAD, LCx, and RCA), and logged false-negative plaque volumes and attenuations for analysis. Patient-level Agatston scores were stratified into standard risk categories: 0 (no coronary artery calcium), 1–99 (mild), 100–399 (moderate), and ≥400 (severe) [13].

### 2.5. Statistical Analysis

All analyses were performed using R (R Core Team, Vienna, Austria; version 4.3.2). TNC served as the reference standard. Continuous variables were assessed visually and using the Shapiro–Wilk test, confirming right-skewed distributions (*p* < 0.001 for all parameters). Therefore, the results are reported as medians with interquartile ranges (IQRs). Paired comparisons between TNC and each VNI series were performed using the Wilcoxon signed-rank test. Reclassification rates and lesion-level sensitivity were calculated with TNC as the reference. The number of patients with at least one FN plaque was recorded, and FN plaques were further stratified by coronary artery and detection pattern. A two-sided *p*-value < 0.05 was considered statistically significant.

## 3. Results

A total of 211 patients were included in the analysis; in total, 181 (85.8%) were male and 30 (14.2%) were female. The median exam-level dose–length product for the coronary CT protocol was 335 mGy.cm (IQR, 278–402).

### 3.1. Quantitative Calcium Burden

Relative to the TNC reference, VNI reconstructions demonstrated a downward bias in coronary calcium burden. The median Agatston score decreased from 35.9 (IQR, 10.3–121.2) on TNC to 23.6 at 55 keV and 22.2 at 60 keV (*p* = 0.0006 and *p* = 0.0003). The total calcified volume likewise declined from 37.8 mm^3^ on TNC to 20.2 mm^3^ (55 keV, *p* = 0.001) and 18.3 mm^3^ (60 keV, *p* < 0.0001).

### 3.2. Risk-Category Agreement

When Agatston scores were stratified into standard clinical categories (0, 1–99, 100–399, ≥400), marked discordance was observed between VNI reconstructions and the TNC reference (Table 1). Although no patient was classified as CAC = 0 on TNC, nearly half were assigned to CAC = 0 on VNI (46.9% on VNI-55 keV; 47.4% on VNI-60 keV). Despite an overall downward bias on VNIs, occasional upward category shifts were observed. For example, VNI-55 keV classified more patients as ≥400 than TNC (15 vs. 13). This pattern is plausible because Agatston scoring is non-linear and depends on both the thresholded lesion area (≥130 HU) and density weighting; thus, modest increases in peak attenuation and/or segmented area above a threshold in a subset of high-burden plaques may yield disproportionate score increases and rare upward reclassification.

At 55 keV, 120/211 patients (56.9%) changed Agatston risk category, predominantly moving downward (107/211; 50.7%), with few upward reclassifications (13/211; 6.2%). At 60 keV, 117/211 patients (55.5%) were reclassified, again mainly moving downward (110/211; 52.1%), with rare upward shifts (7/211; 3.3%).

### 3.3. Plaque-Level Findings

**Figure 1:**
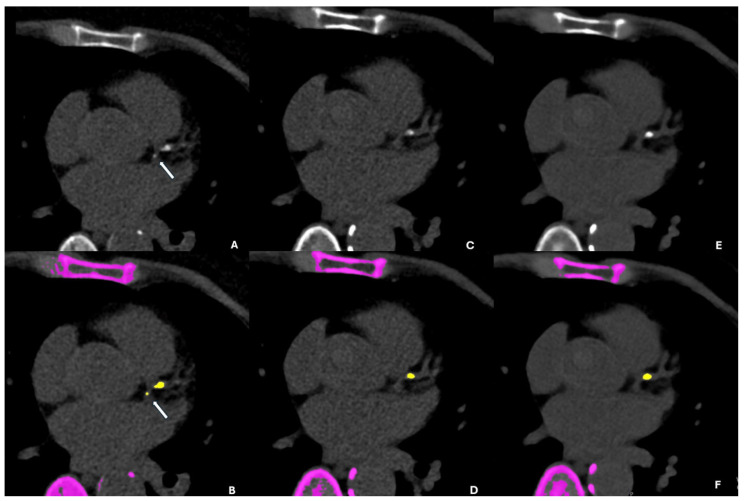
The true non-contrast (TNC), VNI-55 keV, and VNI-60 keV reconstructions and the corresponding automated calcium-scoring outputs. (**A**,**B**): On TNC images and the automated calcium-score output, the white arrow indicates a calcification in the proximal LCx. (**C**,**D**): On VNI-55 keV and its automated output, the proximal LCx calcification seen on TNC is not visualized. (**E**,**F**): Similarly, on VNI-60 keV and its automated output, the proximal LCx calcification evident on TNC is not visualized.

**Figure 2:**
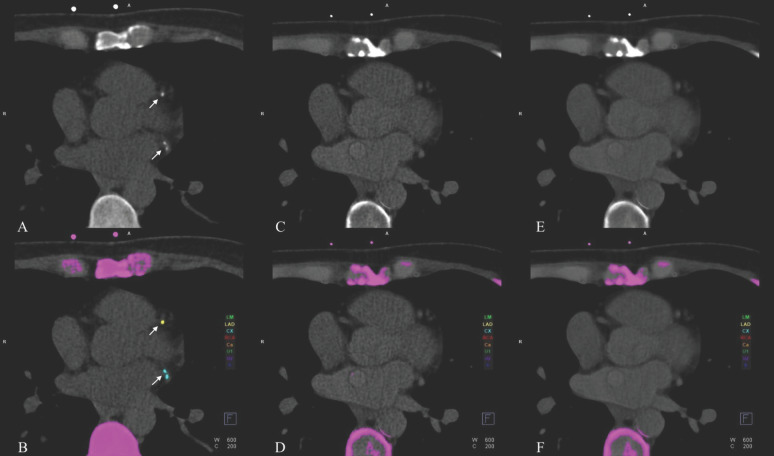
The true non-contrast (TNC), VNI-55 keV, and VNI-60 keV reconstructions and the corresponding automated calcium-scoring outputs. (**A**,**B**): On TNC images and the automated calcium-score output, (**A**,**B**) indicate calcifications in the LAD and LCx. (**C**,**D**): On VNI-55 keV and its automated output, the LAD and LCx calcifications seen on TNC are not visualized. (**E**,**F**): Similarly, on VNI-60 keV and its automated output, the LAD and LCx calcifications evident on TNC are not visualized.

The VNI reconstructions missed at least one plaque in 200 out of 211 patients (94.8%), leaving only 11 patients (5.2%) without a FN plaque. A total of 430 calcified plaques were undetected on at least one VNI reconstruction, namely 427 on VNI-55 and 430 on VNI-60, with substantial overlap. Only three plaques were uniquely missed by VNI-60 keV, and none were uniquely missed by VNI-55 keV. These FN plaques were predominantly small and of low density (Table 2). The majority of FN plaques were located in the LAD and RCA, accounting for approximately 75% of all missed lesions on VNI reconstructions (Table 3). Figure 1 and Figure 2 present side-by-side images of TNC, VNI-55 keV, and VNI-60 keV. Supplementary Materials provide additional examples in DICOM format.

## 4. Discussion

Our study extends prior PCCT work on virtual calcium scoring by emphasizing failure modes that are most consequential for clinical risk stratification and by providing real-world evidence from a tertiary private center in a middle-income Mediterranean country. To enable a focused assessment of false-negative behavior and its potential clinical implications, we restricted the analysis to patients with detectable CAC on the TNC reference (i.e., no true CAC = 0 cases by design). Using a standardized PCCT reconstruction framework (TNC: 70 keV VMI with QIR off; VNI: 55 keV with QIR 1 and 60 keV with QIR 4, with consistent section geometry), we observed a pronounced downward bias on VNI, with a substantial proportion of patients assigned CAC = 0 on VNI despite nonzero CAC on TNC and frequent risk-category reclassification. Importantly, because our sample was restricted to TNC-positive patients, the observed ‘false-zero’ (CAC_VNI = 0 despite CAC_TNC > 0) frequency quantifies misclassification under the tested reconstruction settings, not the real-world prevalence of CAC = 0 on VNI.

A key contribution of this work is plaque-level adjudication at scale, linking patient-level reclassification to lesion characteristics. False-negative findings were common, and missed lesions were predominantly small and of low density, consistent with prior reports that VNI-based scoring is density-dependent and particularly vulnerable near the 130 HU threshold and at very low calcium burden [4,7]. Accordingly, these data suggest vulnerability near the CAC = 0 threshold under the tested reconstruction settings; the implications for downstream management and prognosis require prospective validation.

Although this study was not designed to assess downstream clinical decisions or outcomes, misclassification at CAC = 0 may matter because CAC = 0 is often treated as a binary decision point in primary prevention and risk communication. In threshold-sensitive settings, a false-zero result could lead to the underestimation of calcified plaque burden during clinician–patient counseling. These statements are hypothesis-generating and should be interpreted as potential implications rather than demonstrated effects in our dataset.

Sharma et al. found that VNC underestimated and VNI overestimated CACS versus TNC (median CACS: TNC 27.8 vs. VNC 8.5 vs. VNI 72.2) and reported lower overall misclassification for VNI than VNC (32% vs. 55%). Their TNC reference was reconstructed as 70 keV VMI (3 mm slice thickness/1.5 mm increment) with a Qr36 kernel and iterative reconstruction strength 3, and the VNC/VNI series were reconstructed with matched parameters (also 70 keV VMI, 3.0 mm/1.5 mm, Qr36) to maximize comparability [3]. False-zero misclassification was present but uncommon (7.8%), and their sample included substantial representation across higher CAC categories rather than being dominated by borderline disease. In contrast, we intentionally evaluated lower-keV VNI reconstructions (55 and 60 keV) based on published PCCT optimization work [11]. As this optimization research shows that virtual-reconstruction CAC scores generally decrease with increasing keV (i.e., a lower keV tends to increase detectability and scores), our higher downward reclassification/false-zero rate is unlikely to be explained by keV selection alone. A more plausible explanation is sample composition enriched for relatively low-burden, threshold-sensitive plaques near the 130 HU detection cutoff, where small HU/noise shifts can disproportionately convert positive findings into sub-threshold results.

Vecsey-Nagy et al. reported that 18.3% of patients were falsely labeled CACS = 0 on VNI and that higher tube voltage (140 kVp) and higher CAC density independently reduced deviation from TNC [7]. However, the ratio of patients falsely labeled as CAC = 0 was much higher in our sample for both 55 keV and 60 keV VNI reconstruction. Our FN lesions were predominantly small and of low density, aligning with their conclusion that small/low-density foci are most vulnerable to VNI misclassification and that acquisition energy/density materially influence agreement [7].

Using calcium-preserving VNI from late-enhancement PCCT, Mergen et al. demonstrated near-identity with TNC for AVC/MAC and very high agreement for CAC at optimized keV/QIR (e.g., κ ≈ 0.97 for CAC), suggesting that when the algorithm and task are well matched, CACS can be reliable without a dedicated TNC [14]. Their VNC images were reconstructed at 60, 70, 80, and 90 keV, respectively, applying QIR with strengths of 2, 3, and 4, respectively. Our discrepant results, therefore, likely reflect a different reconstruction context and a lesion mix enriched for small/low-density plaques [14].

Braun et al. showed that PCCT-based VNI can meet key prerequisites—effective iodine removal, low noise, and reliable calcium preservation. Their key settings include VMI at 70 keV, kernel Qr36, and 3.0 mm thickness/1.5 mm increment, with QIR off for their conventional VNC, while their PureCalcium/VNI reconstruction uses QIRs of 3 and 4, respectively. The conventional algorithm (VNCConv) consistently underestimated calcium, whereas the calcium-preserving algorithm (VNI/PureCalcium) closely matched TNC (r^2^ > 0.93) for Agatston and volume, supporting the use of VNI as a potential TNC substitute that reduces dose, time, and cost [15]. In contrast, in our larger sample, low-keV VNI (55–60 keV) underestimated calcium burden, frequently reclassified patients to lower risk categories, and missed small, low-density plaques, leading to false-zero assignments around the threshold.

Haag et al. studied 170 patients and reported excellent correlation between true non-contrast (TNC) and PureCalcium-derived calcium scores (ICC = 0.98), with no significant difference in the median Agatston score (*p* = 0.99). However, Bland–Altman analysis showed wide limits of agreement, indicating limited patient-level concordance. PureCalcium correctly classified 74% of patients (κ = 0.88), but misclassification clustered at the zero-score threshold: among TNC-positive patients, 14/111 (12.6%) were classified as P0 on PureCalcium (i.e., “false-zero” behavior), highlighting vulnerability near CAC = 0 [4]. Their sample was also low-burden-enriched (P0 + P1 = 76.5%), which increases susceptibility to threshold-sensitive reclassification. Methodologically, reconstructions were performed in 70 keV VMI space, but the series were not perfectly matched: TNC was reconstructed at 70 keV using Qr36 with QIR off (3.0 mm thickness/1.5 mm increment), whereas PureCalcium used QIR level 3 with a different kernel (Qr40) and thinner sections (2.0 mm/1.5 mm increment).

In our larger sample, the same failure mode was substantially more pronounced: approximately 50% of examinations were labeled CAC = 0 on VNI despite detectable calcium on TNC, and 95% had at least one false-negative lesion—typically small, low-density plaques. We note that the thinner section thickness used for PureCalcium in their study may increase sensitivity for small calcifications, which could partly mitigate false-zero classification compared with the TNC-matched thicker-section reconstructions used in our study.

Kaldas et al. reported excellent VNC–TNC concordance on PCCT (ICC 0.97–0.99). Misclassification was uncommon when comparing TNC 3 × 1.5 mm to VNC (κ = 0.94), and overall categorical agreement remained high (κ 0.83–0.85) [16]. Their protocol used a deliberately standardized reconstruction framework—70 keV monoenergetic images with QIR strength 2, a 512 × 512 matrix, a QR36 kernel, and 3.0 mm sections reconstructed with both 3.0 mm and 1.5 mm increments—to isolate the effect of slice increment while keeping other parameters stable. However, they still observed statistically significant differences in mean calcium scores and nonzero misclassification, underscoring that the reconstruction method can influence CACS even under controlled settings. Importantly, their analysis excluded patients with CACS = 0 and had a relatively higher calcium-burden distribution, limiting direct benchmarking of the clinically sensitive “false-zero” failure mode. By contrast, in our larger, low-burden-enriched sample using low-keV VNI, we observed substantial underestimation with frequent downward reclassification, with nearly half of the examinations labeled CAC = 0 despite detectable calcium on TNC.

Fink et al. showed that a targeted safety-net VNI, i.e., 1.0 mm, Qr44, QIR 2, 55 keV, 110–120 HU, with a background-Agatston check, recovers ~80–90% of subtle calcifications missed by standard VNI. In view of our systematic underestimation with routine VNI, such a safety-net reconstruction represents a plausible mitigation strategy; however, it was outside the scope of the present study and was not empirically evaluated in our sample. Therefore, this discussion is hypothesis-generating and should not be interpreted as prescriptive or as a protocol recommendation based on our data. At present, such strategies do not replace TNC as the reference standard but—if prospectively validated under standardized conditions—may reduce inappropriate de-risking and provide a standardized mitigation option in selected CCTA-derived workflows.

Several limitations should be acknowledged. First, this was a single-center, retrospective study performed on one PCCT platform and a single vendor software chain (PureCalcium) using prespecified reconstruction choices (TNC: 70 keV VMI with QIR off; VNI: 55 keV with QIR 1 and 60 keV with QIR 4; fixed kernel and slice geometry). Accordingly, our findings may not generalize to other vendors, scanner generations, software versions, reconstruction kernels/QIR strengths, monoenergetic settings, or postprocessing pipelines. Second, because we excluded patients with CAC = 0 on the TNC reference by design, the reported “false-zero” proportion reflects misclassification within a TNC-positive sample and should not be interpreted as a population-level prevalence estimate for CAC = 0 on VNI in routine practice. Third, we did not perform HU recalibration for VNI, nor did we systematically evaluate alternative Agatston thresholds or mitigation (“safety-net”) strategies (e.g., thinner sections, higher-detail kernels, higher QIR strengths, or additional monoenergetic levels), any of which could reduce underestimation in selected settings. Fourth, plaque-level adjudication was performed by a single expert reader; inter-reader agreement was not assessed. Because missed lesions were predominantly small and/or of low density and near the 130 HU threshold, plaque-level false-negative classification and lesion-level sensitivity estimates may be reader-dependent and should be interpreted cautiously. In a similar vein, because plaque identification began from automated calcium-scoring outputs and involved reader confirmation/correction as needed, the extent of manual adjustment may influence FN plaque counts and lesion-level sensitivity estimates, particularly for borderline calcifications near the 130 HU threshold. We did not perform a formal subset re-read for internal consistency; therefore, intra-reader repeatability could not be quantified in this dataset. Fifth, our sample was enriched for low-burden calcification, which may accentuate threshold sensitivity and should be considered when extrapolating these findings to populations with higher calcium burden. Finally, we did not capture downstream decision-making, preventive therapy initiation, follow-up imaging, or cardiovascular events; therefore, we cannot determine whether VNI–TNC discordance translates into differences in management or prognosis.

## 5. Conclusions

In a large single-center sample with low-burden calcification from a private tertiary center in a middle-income Mediterranean country, low-keV CCTA-derived VNI (55–60 keV) showed lower CACS than the TNC reference and was associated with frequent downward risk-category shifts, including a substantial number of zero-score assignments despite nonzero CAC on TNC. These findings suggest that, in cohorts with low-burden calcification, VNI-based scoring may be vulnerable near the CAC = 0 threshold, as well as that additional mitigation strategies (e.g., alternative reconstructions or “safety-net” approaches) may be needed before VNI can be relied upon for de-risking decisions in routine workflows.

## Figures and Tables

**Table 1:** The distribution of coronary artery calcium risk categories across reconstructions.

Risk Category	TNC	VNI-55 keV	VNI-60 keV
0	0 (0%)	99 (46.9%)	100 (47.4%)
1–99	146 (69.2%)	45 (21.3%)	50 (23.7%)
100–399	52 (24.6%)	52 (24.6%)	48 (22.7%)
≥400	13 (6.2%)	15 (7.1%)	13 (6.2%)

TNC = true non-contrast; VNI = virtual non-iodine.

**Table 2:** The characteristics of plaques missed on virtual non-iodine reconstructions.

Sequence	*n* Plaques	Volume (mm^3^) Median [IQR]	Agatston Score Median [IQR]
VNI-55 keV	427	8.3 [4.9–15.0]	6.4 [3.0–13.4]
VNI-60 keV	430	8.4 [4.9–15.1]	6.4 [3.0–13.8]

**Table 3:** The arterial distribution of false-negative plaques.

Coronary Segment	VNI-55 keV	VNI-60 keV
LAD	218 (51.1%)	219 (50.9%)
RCA	104 (24.4%)	105 (24.4%)
LCx	78 (18.3%)	79 (18.4%)
LM	27 (6.3%)	27 (6.3%)
Total	427 (100%)	430 (100%)

VNI = virtual non-iodine (PureCalcium); LAD = left anterior descending artery; RCA = right coronary artery; LCx = left circumflex artery; LM = left main coronary artery.

## Data Availability

The data presented in this study are available on request from the corresponding author. The data are not publicly available due to patient privacy.

## Supplementary Materials

The following supporting information can be downloaded at: https://www.mdpi.com/article/10.3390/diagnostics16040599/s1, Additional example cases are provided as DICOM files.

## Abbreviations

The following abbreviations are used in this manuscript:

CAC: coronary artery calcium; CACS: coronary artery calcium score; CCTA: coronary computed tomography angiography; CI: confidence interval; CT: computed tomography; DICOM: Digital Imaging and Communications in Medicine; DLP: dose–length product; ECG: electrocardiography; FN: false negative; HR: heart rate; HU: Hounsfield unit; ICC: intraclass correlation coefficient; IQR: interquartile range; keV: kiloelectron volt; kVp: kilovoltage peak; LAD: left anterior descending (coronary) artery; LCx: left circumflex (coronary) artery; LM: left main (coronary) artery; mAs: milliampere-second; mGy: milligray; NRI: net reclassification improvement; PCCT: photon-counting computed tomography; QIR: quantum iterative reconstruction; RCA: right coronary artery; ROI: region of interest; SD: standard deviation; TNC: true non-contrast (scan/series); VMI: virtual monoenergetic image; VNI: virtual non-iodine.
