# Supplementary material for: Virtual Non-Iodine Coronary Calcium Scoring on Photon-Counting CT: Patient- and Plaque-Level Analysis
Source: Diagnostics (Basel). 2026 Feb 17;16(4):599. doi: 10.3390/diagnostics16040599 (PMC12939015; doi:10.3390/diagnostics16040599)
Supplement: Supplementary file 1 [file diagnostics-16-00599-s001.zip › Samples/Patient 1/60 KEV/syngo_fV/readme.rtf]

syngo fastView

© 2016 by Siemens Healthcare GmbH, Erlangen, Germany.
All rights reserved.
       
Internet:
	http://www.syngo.com
	
END-USER LICENSE AGREEMENT FOR THIS SOFTWARE

IMPORTANT-READ CAREFULLY: This End-User License
Agreement is a legal agreement between you (either an
individual or a single entity) and Siemens Healthcare for 
this software product identified above, which includes
computer software and associated media and printed materials,
and may include "online" or electronic documentation ("SOFTWARE
PRODUCT" or "SOFTWARE"). By installing, copying, or otherwise
using the SOFTWARE PRODUCT, you agree to be bound by the terms
of this license agreement. If you do not agree to the terms of 
this agreement, do not install, copy, or use the SOFTWARE PRODUCT.

SOFTWARE PRODUCT LICENSE
The SOFTWARE PRODUCT is protected by copyright laws and
international copyright treaties, as well as other intellectual
property laws and treaties. The SOFTWARE PRODUCT is licensed,
not sold.

1. GRANT OF LICENSE. This license agreement grants you the following
non-exclusive right:

•	Software Product. You may install and use the enclosed 
SOFTWARE PRODUCT on your computer.
•	syngo fastView is not a medical device and therefore not 
permitted for diagnostic use.
For additional information please refer to the online help.
•	Copies of clinical images together with syngo fastView can 
       be handed over to patients on CD or DVD for private use only.

Siemens Healthcare reserves all rights not expressly granted to you.


2.  DESCRIPTION OF OTHER RIGHTS AND LIMITATIONS.
•	Limitations on Reverse-Engineering, Decompilation, and
Disassembly. You may not reverse- engineer, decompile, or
disassemble the SOFTWARE PRODUCT.

•	Rental. You may not rent or lease the SOFTWARE PRODUCT.

•	Termination. Without prejudice to any other rights, Siemens
Medical Solutions may terminate this license agreement if you fail 
to comply with the terms and conditions of this agreement. In such
event, you must destroy all copies of the SOFTWARE PRODUCT
and all of its component parts.


NO WARRANTIES. To the maximum extent permitted by applicable
law, Siemens Healthcare expressly disclaims any warranty for the 
SOFTWARE PRODUCT. The SOFTWARE PRODUCT and any related 
documentation are provided "as is" without warranty of any kind, either 
express or implied, including, without limitation, the implied warranties of 
merchantability or fitness for a particular purpose. The entire risk arising 
out of use or performance of the SOFTWARE PRODUCT remains with you.

NO LIABILITY FOR CONSEQUENTIAL DAMAGES. 
To the maximum extent permitted by applicable law, in no event 
shall Siemens Healthcare or its suppliers be liable for any 
damages whatsoever (including, without limitation, damages for loss of 
business profit, business interruption, loss of business information, or 
any other pecuniary loss) arising out of the use of, or inability to
use, this product, even if Siemens Healthcare has been 
advised of the possibility of such damages. Because some
states/jurisdictions do not allow the exclusion or limitation of
liability for consequential or incidental damages, the above
limitation may not apply to you.


syngo   is a registered trademark owned by Siemens Healthcare GmbH.
